# Supplementary material for: Constitutive Models for Active Skeletal Muscle: Review, Comparison, and Application in a Novel Continuum Shoulder Model
Source: Int J Numer Method Biomed Eng. 2025 Apr 23;41(4):e70036. doi: 10.1002/cnm.70036 (PMC12017808; doi:10.1002/cnm.70036)
Supplement: Supplementary file 1 — Data S1. Supporting Information. [file CNM-41-e70036-s001.pdf]

Supplementary material

S1 Elasticity tensors

S1.1 Active stress approach (ASE)

Considering the isochoric and volumetric contributions in the strain-energy function in Eq. (8), the elasticity tensor is computed to

$$\mathbb{C} = \mathbb{C}_{\text{iso}} + \mathbb{C}_{\text{vol}}. \tag{S1}$$

The volumetric part reads

$$\mathbb{C}_{\text{vol}} = K\left(\mathbf{C}^{-1} \otimes \mathbf{C}^{-1} + 2\ln J \frac{\partial \mathbf{C}^{-1}}{\partial \mathbf{C}}\right) \tag{S2}$$

with

$$\frac{\partial \mathbf{C}^{-1}}{\partial \mathbf{C}}_{ijkl} = -\tfrac{1}{2}\left(C_{ik}^{-1}C_{jl}^{-1} + C_{il}^{-1}C_{jk}^{-1}\right). \tag{S3}$$

The isochoric part is computed using the ficticious elasticity tensor $\bar{\mathbb{C}} = 2J^{-4/3}\frac{\partial \bar{\mathbf{S}}}{\partial \bar{\mathbf{C}}}$ and the modified projection tensor $\tilde{\mathbb{P}} = \mathbf{C}^{-1} \odot \mathbf{C}^{-1} - \frac{1}{3}\mathbf{C}^{-1} \otimes \mathbf{C}^{-1}$ according to the relation

$$\mathbb{C}_{\text{iso}} = \mathbb{P} : \bar{\mathbb{C}} : \mathbb{P}^T + \tfrac{2}{3}J^{-2/3}\left(\bar{\mathbf{S}} : \bar{\mathbf{C}}\right)\tilde{\mathbb{P}} - \tfrac{2}{3}\left(\mathbf{C}^{-1} \otimes \mathbf{S}_{\text{iso}} + \mathbf{S}_{\text{iso}} \otimes \mathbf{C}^{-1}\right). \tag{S4}$$

According to [1, p.272], the ficticious elasticity tensor $\bar{\mathbb{C}}$ for a transversely isotropic material model reads

$$\begin{aligned}\bar{\mathbb{C}} = J^{-4/3}\Bigg\{&\bar{\delta}_1\left(\mathbf{I} \otimes \mathbf{I}\right) + \bar{\delta}_5\left(\mathbf{I} \otimes \mathbf{M} + \mathbf{M} \otimes \mathbf{I}\right) + \bar{\delta}_7\left(\mathbf{M} \otimes \mathbf{M}\right) + \bar{\delta}_8\left(\mathbf{I} \otimes \frac{\partial \bar{I}_5}{\partial \bar{\mathbf{C}}} + \frac{\partial \bar{I}_5}{\partial \bar{\mathbf{C}}} \otimes \mathbf{I}\right)\\ &+ \bar{\delta}_{10}\left(\frac{\partial \bar{I}_5}{\partial \bar{\mathbf{C}}} \otimes \frac{\partial \bar{I}_5}{\partial \bar{\mathbf{C}}}\right) + \bar{\delta}_{11}\left(\mathbf{M} \otimes \frac{\partial \bar{I}_5}{\partial \bar{\mathbf{C}}} + \frac{\partial \bar{I}_5}{\partial \bar{\mathbf{C}}} \otimes \mathbf{M}\right) + \bar{\delta}_{12}\frac{\partial^2 \bar{I}_5}{\partial \bar{\mathbf{C}}^2}\Bigg\}\end{aligned} \tag{S5}$$

with the pre-factors

$$\bar{\delta}_1 = -2G_2A_3\bar{I}_4, \qquad \bar{\delta}_5 = 2G_2\left(A_2 - A_3\left(\bar{I}_1 - A_1\right)\right), \qquad \bar{\delta}_8 = 2G_2A_3,$$
$$\bar{\delta}_7 = 24G_1\bar{I}_5\bar{I}_4^{-4} - 2G_2\left(A_3\left(\bar{I}_1 - A_1\right)^2 + A_2\left(2\bar{I}_1 - 3A_1\right)\right)\bar{I}_4^{-1} - 2\bar{I}_4^{-2}\left(\sigma_{\text{f}}^{\text{tot}} - \tfrac{\partial \sigma_{\text{f}}^{\text{tot}}}{\partial \bar{\lambda}}\bar{I}_4\right),$$
$$\bar{\delta}_{10} = -2G_2A_3\bar{I}_4^{-1}, \qquad \bar{\delta}_{11} = 2G_2\left(A_2 + A_3\left(\bar{I}_1 - A_1\right)\right)\bar{I}_4^{-1} - 8G_1\bar{I}_4^{-3}, \qquad \bar{\delta}_{12} = 4G_1\bar{I}_4^{-2} - 4G_2A_2,$$

and the derivatives

$$\frac{\partial \bar{I}_5}{\partial \bar{\mathbf{C}}} = \mathbf{M}\bar{\mathbf{C}} + \bar{\mathbf{C}}\mathbf{M} \qquad \text{and} \qquad \frac{\partial^2 \bar{I}_5}{\partial \bar{\mathbf{C}}^2} = \mathbf{I} \otimes \mathbf{M} + \mathbf{M} \otimes \mathbf{I}.$$

Differentiating $\sigma_{\text{f}}^{\text{tot}}$ with respect to the fiber stretch results in the derivative

$$\frac{\partial \sigma_{\text{f}}^{\text{tot}}}{\partial \bar{\lambda}} = \sigma_{\max}\tfrac{1}{\lambda_{\text{opt}}^{\text{a}}}af_{\text{t}}^{\tanh}\frac{\partial \bar{\lambda} f_{\xi}^{\text{a}}}{\partial \bar{\lambda}} + \sigma_{\max}\frac{\partial \bar{\lambda} f_{\xi}^{\text{p}}}{\partial \bar{\lambda}} \tag{S6}$$

with the terms

$$\frac{\partial \bar{\lambda} f_{\xi}^{\text{p}}}{\partial \bar{\lambda}} = \begin{cases} 2D_3\bar{\lambda} + D_4 & \text{if } \bar{\lambda} \geq \lambda_* \\ \bar{\lambda}D_1D_2\mathrm{e}^{D_2(\bar{\lambda}-1)} + D_1\left(\mathrm{e}^{D_2(\bar{\lambda}-1)} - 1\right) & \text{if } \lambda_* > \bar{\lambda} > 1 \\ 0 & \text{if } 1 \geq \bar{\lambda}, \end{cases} \tag{S7}$$

$$\frac{\partial \bar{\lambda} f_{\xi}^{\text{a}}}{\partial \bar{\lambda}} = \begin{cases} 27\left(\frac{\bar{\lambda}}{\lambda_{\text{opt}}^{\text{a}}}\right)^2 - 14.4\frac{\bar{\lambda}}{\lambda_{\text{opt}}^{\text{a}}} + 1.44 & \text{if } \bar{\lambda} \leq 0.6\lambda_{\text{opt}}^{\text{a}} \\ -12\left(\frac{\bar{\lambda}}{\lambda_{\text{opt}}^{\text{a}}}\right)^2 + 16\frac{\bar{\lambda}}{\lambda_{\text{opt}}^{\text{a}}} - 3 & \text{if } 0.6\lambda_{\text{opt}}^{\text{a}} < \bar{\lambda} < 1.4\lambda_{\text{opt}}^{\text{a}} \\ 27\left(\frac{\bar{\lambda}}{\lambda_{\text{opt}}^{\text{a}}}\right)^2 - 57.6\frac{\bar{\lambda}}{\lambda_{\text{opt}}^{\text{a}}} + 23.04 & \text{if } \bar{\lambda} \geq 1.4\lambda_{\text{opt}}^{\text{a}}. \end{cases} \tag{S8}$$

S1.2 Generalized active strain approach (GASA)

With the strain-energy function in Eq. (16), the material tangent $\mathbb{C}$ reads, as published in [2],

$$\begin{aligned}\mathbb{C} = 2\frac{\partial \mathbf{S}}{\partial \mathbf{C}} = \gamma \Bigg[&\delta_0 \left(\tilde{\mathbf{L}} + \omega_\mathrm{a}\mathbf{M}\right) \otimes \left(\tilde{\mathbf{L}} + \omega_\mathrm{a}\mathbf{M}\right) + \delta_1 \left(\tilde{\mathbf{L}} + \frac{1+\omega_\mathrm{a}\alpha\lambda^2}{\alpha\lambda^2}\mathbf{M}\right) \otimes \frac{\partial \omega_\mathrm{a}}{\partial \mathbf{C}} \\ &+ \delta_2 \left(\mathbf{C}^{-1}\tilde{\mathbf{L}}\mathbf{C}^{-1} \otimes \mathbf{C}^{-1}\tilde{\mathbf{L}}\mathbf{C}^{-1}\right) + \delta_3 \left(\mathbf{C}^{-1} \otimes \mathbf{C}^{-1}\tilde{\mathbf{L}}\mathbf{C}^{-1} + \mathbf{C}^{-1}\tilde{\mathbf{L}}\mathbf{C}^{-1} \otimes \mathbf{C}^{-1}\right) \\ &+ \delta_4 \mathbf{C}^{-1} \otimes \mathbf{C}^{-1} + \delta_5 \frac{\partial \mathbf{C}^{-1}}{\partial \mathbf{C}} + \delta_6 \frac{\partial \mathbf{C}^{-1}\tilde{\mathbf{L}}\mathbf{C}^{-1}}{\partial \mathbf{C}} \Bigg]\end{aligned} \tag{S9}$$

with the scalar pre-factors

$$\begin{gathered}\delta_0 = \alpha \mathrm{e}^{\alpha(\tilde{I}-1)}, \qquad \delta_1 = \lambda^2\alpha \mathrm{e}^{\alpha(\tilde{I}-1)}, \qquad \delta_2 = \beta \mathrm{e}^{\beta(\tilde{J}-1)} \det(\mathbf{C})^2, \\ \delta_3 = -(\beta\tilde{J}+1)\mathrm{e}^{\beta(\tilde{J}-1)} \det(\mathbf{C}), \qquad \delta_4 = (\beta\tilde{J}+1)\mathrm{e}^{\beta(\tilde{J}-1)} + \kappa \det(\mathbf{C})^{-\kappa}, \\ \delta_5 = \tilde{J}\mathrm{e}^{\beta(\tilde{J}-1)} - \det(\mathbf{C})^{-\kappa}, \qquad \delta_6 = -\mathrm{e}^{\beta(\tilde{J}-1)} \det(\mathbf{C}).\end{gathered}$$

While the derivative $\frac{\partial \mathbf{C}^{-1}}{\partial \mathbf{C}}$ is given by Eq. (S3), the derivative $\frac{\partial \mathbf{C}^{-1}\tilde{\mathbf{L}}\mathbf{C}^{-1}}{\partial \mathbf{C}}$ is computed to

$$\begin{aligned}\left.\frac{\partial \mathbf{C}^{-1}\tilde{\mathbf{L}}\mathbf{C}^{-1}}{\partial \mathbf{C}}\right|_{ijkl} = &-\tfrac{1}{2}\left(C^{-1}_{ik}C^{-1}_{jm}\tilde{L}_{mn}C^{-1}_{nl} + C^{-1}_{il}C^{-1}_{jm}\tilde{L}_{mn}C^{-1}_{nk}\right) \\ &-\tfrac{1}{2}\left(C^{-1}_{jk}C^{-1}_{im}\tilde{L}_{mn}C^{-1}_{nl} + C^{-1}_{jl}C^{-1}_{im}\tilde{L}_{mn}C^{-1}_{nk}\right).\end{aligned} \tag{S10}$$

The derivative of the activation level $\frac{\partial \omega_\mathrm{a}}{\partial \mathbf{C}}$ is obtained as

$$\frac{\partial \omega_\mathrm{a}}{\partial \mathbf{C}} = \frac{1}{2\lambda}\frac{\partial \omega_\mathrm{a}}{\partial \lambda}\mathbf{M} \tag{S11}$$

with

$$\frac{\partial \omega_\mathrm{a}}{\partial \lambda} = \begin{cases} 0 & \text{if } P_\mathrm{a} = 0 \\ \frac{1}{\alpha\lambda^2}\frac{\mathrm{d}W_0(\chi^*)}{\mathrm{d}\lambda} - \frac{2W_0(\chi^*)}{\alpha\lambda^3} - \frac{1}{2\lambda}\tilde{I}''_\mathrm{p} + \frac{1}{2\lambda^2}\tilde{I}'_\mathrm{p} & \text{else.}\end{cases} \tag{S12}$$

While $\tilde{I}'_\mathrm{p}$, the first derivative of the passive part of the first generalized invariant $\tilde{I}_\mathrm{p}$ with respect to λ, is given by Eq. (A5), the second derivative $\tilde{I}''_\mathrm{p}$ reads

$$\tilde{I}''_\mathrm{p} = \frac{\partial^2 \tilde{I}_\mathrm{p}}{\partial \lambda^2} = \tfrac{4}{3}\omega_0\left(\lambda^{-3} - 1\right) + 2. \tag{S13}$$

Differentiation of the Lambert W function with respect to λ results in

$$\frac{\mathrm{d}W_0(\chi^*)}{\mathrm{d}\lambda} = \frac{1}{(1+W_0)\,\mathrm{e}^{W_0}}\frac{\mathrm{d}\chi^*}{\mathrm{d}\lambda}.$$

whereby the latter factor is computed to

$$\begin{aligned}\frac{\mathrm{d}\chi^*}{\mathrm{d}\lambda} = &\tfrac{2\alpha}{\gamma}\mathrm{e}^{\frac{\alpha}{2}(2-2\tilde{I}_\mathrm{p}+\lambda\tilde{I}'_\mathrm{p})}\left[P_\mathrm{a} + \lambda\frac{\mathrm{d}P_\mathrm{a}}{\mathrm{d}\lambda} + \tfrac{\alpha}{2}P_\mathrm{a}\lambda\left(\lambda\tilde{I}''_\mathrm{p} - \tilde{I}'_\mathrm{p}\right)\right] \\ &+ \tfrac{2\alpha+\alpha^2}{4}\mathrm{e}^{\frac{\alpha}{2}\lambda\tilde{I}'_\mathrm{p}}\left(\lambda\tilde{I}''_\mathrm{p} + \tilde{I}'_\mathrm{p}\right).\end{aligned} \tag{S14}$$

Under the assumption that the force-velocity dependency f_v is neglected, the first derivative of P_a with respect to λ is calculated as

$$\frac{\mathrm{d}P_\mathrm{a}}{\mathrm{d}\lambda} = N_\mathrm{a}\sum_{i=1}^{n_\mathrm{MU}} \rho_i F^i_\mathrm{t}\frac{\mathrm{d}f_\xi}{\mathrm{d}\lambda} \tag{S15}$$

with

$$\frac{\mathrm{d}f_\xi}{\mathrm{d}\lambda} = \begin{cases} \frac{(\lambda_\mathrm{min}-\lambda)^2-(\lambda_\mathrm{min}-\lambda_\mathrm{opt})^2}{2(\lambda_\mathrm{min}-\lambda_\mathrm{opt})^3}\exp\left[\frac{(2\lambda_\mathrm{min}-\lambda-\lambda_\mathrm{opt})(\lambda-\lambda_\mathrm{opt})}{2(\lambda_\mathrm{min}-\lambda_\mathrm{opt})^2}\right] & \text{if } \lambda > \lambda_\mathrm{min} \\ 0 & \text{if } \lambda \leqslant \lambda_\mathrm{min}.\end{cases} \tag{S16}$$

S1.3 Active strain approach (ASA)

Based on the strain-energy function in Eq. (23), the elasticity tensor $\mathbb{C}$ contains an elastic and volumetric contribution according to

$$\mathbb{C} = \mathbb{C}_{\mathrm{e}} + \mathbb{C}_{\mathrm{vol}}. \tag{S17}$$

Considering the derivative $\frac{\partial \mathbf{C}^{-1}}{\partial \mathbf{C}}$ in Eq. (S3), the volumetric component can be computed to

$$\mathbb{C}_{\mathrm{vol}} = \gamma \det(\mathbf{C})^{-\kappa} \left(\kappa \mathbf{C}^{-1} \otimes \mathbf{C}^{-1} - \frac{\partial \mathbf{C}^{-1}}{\partial \mathbf{C}} \right). \tag{S18}$$

For simplicity reasons, we use index notation for the complex derivation of the elastic contribution $\mathbb{C}_{\mathrm{e}}$. Its components are given as

$$\begin{aligned}
\mathbb{C}_{\mathrm{e},ijkl} = 4 \det(\mathbf{F}_{\mathrm{a}}) \Bigg\{ & \left(\frac{\partial \mathbf{F}_{\mathrm{a}}^{-1}}{\partial \omega_{\mathrm{a}}} \mathbf{C} \frac{\partial \mathbf{F}_{\mathrm{a}}^{-1}}{\partial \omega_{\mathrm{a}}} + {\mathbf{F}_{\mathrm{a}}}^{-1} \mathbf{C} \frac{\partial^2 \mathbf{F}_{\mathrm{a}}^{-1}}{\partial \omega_{\mathrm{a}}^2} \right)_{rs} \mathbf{S}_{\mathrm{e}rs} \frac{\partial \omega_{\mathrm{a}}}{\partial \mathbf{C}}_{ij} \frac{\partial \omega_{\mathrm{a}}}{\partial \mathbf{C}}_{kl} \\
& + \left({\mathbf{F}_{\mathrm{a}}}^{-1} \mathbf{C} \frac{\partial \mathbf{F}_{\mathrm{a}}^{-1}}{\partial \omega_{\mathrm{a}}} \right)_{rs} \mathbf{S}_{\mathrm{e}rs} \frac{\partial^2 \omega_{\mathrm{a}}}{\partial \mathbf{C}^2}_{ijkl} \\
& + \tfrac{1}{2} \frac{\partial \omega_{\mathrm{a}}}{\partial \mathbf{C}}_{ij} \left(\frac{\partial \mathbf{F}_{\mathrm{a}}^{-1}}{\partial \omega_{\mathrm{a}}} \mathbf{S}_{\mathrm{e}} {\mathbf{F}_{\mathrm{a}}}^{-1} + {\mathbf{F}_{\mathrm{a}}}^{-1} \mathbf{S}_{\mathrm{e}} \frac{\partial \mathbf{F}_{\mathrm{a}}^{-1}}{\partial \omega_{\mathrm{a}}} \right)_{kl} \\
& + \tfrac{1}{2} \left(\frac{\partial \mathbf{F}_{\mathrm{a}}^{-1}}{\partial \omega_{\mathrm{a}}} \mathbf{S}_{\mathrm{e}} {\mathbf{F}_{\mathrm{a}}}^{-1} + {\mathbf{F}_{\mathrm{a}}}^{-1} \mathbf{S}_{\mathrm{e}} \frac{\partial \mathbf{F}_{\mathrm{a}}^{-1}}{\partial \omega_{\mathrm{a}}} \right)_{ij} \frac{\partial \omega_{\mathrm{a}}}{\partial \mathbf{C}}_{kl} \\
& + 2 \left({\mathbf{F}_{\mathrm{a}}}^{-1} \mathbf{C} \frac{\partial \mathbf{F}_{\mathrm{a}}^{-1}}{\partial \omega_{\mathrm{a}}} \right)_{rs} \frac{\partial \mathbf{S}_{\mathrm{e}}}{\partial \mathbf{C}_{\mathrm{e}}}_{rstu} \left({\mathbf{F}_{\mathrm{a}}}^{-1} \mathbf{C} \frac{\partial \mathbf{F}_{\mathrm{a}}^{-1}}{\partial \omega_{\mathrm{a}}} \right)_{tu} \frac{\partial \omega_{\mathrm{a}}}{\partial \mathbf{C}}_{ij} \frac{\partial \omega_{\mathrm{a}}}{\partial \mathbf{C}}_{kl} \\
& + \frac{\partial \omega_{\mathrm{a}}}{\partial \mathbf{C}}_{ij} \mathbf{F}_{\mathrm{a}}{}^{-1}_{kt} \left({\mathbf{F}_{\mathrm{a}}}^{-1} \mathbf{C} \frac{\partial \mathbf{F}_{\mathrm{a}}^{-1}}{\partial \omega_{\mathrm{a}}} \right)_{rs} \frac{\partial \mathbf{S}_{\mathrm{e}}}{\partial \mathbf{C}_{\mathrm{e}}}_{rstu} \mathbf{F}_{\mathrm{a}}{}^{-1}_{ul} \\
& + \mathbf{F}_{\mathrm{a}}{}^{-1}_{ir} \frac{\partial \mathbf{S}_{\mathrm{e}}}{\partial \mathbf{C}_{\mathrm{e}}}_{rstu} \left({\mathbf{F}_{\mathrm{a}}}^{-1} \mathbf{C} \frac{\partial \mathbf{F}_{\mathrm{a}}^{-1}}{\partial \omega_{\mathrm{a}}} \right)_{tu} \mathbf{F}_{\mathrm{a}}{}^{-1}_{sj} \frac{\partial \omega_{\mathrm{a}}}{\partial \mathbf{C}}_{kl} \\
& + \tfrac{1}{2} \mathbf{F}_{\mathrm{a}}{}^{-1}_{ir} \mathbf{F}_{\mathrm{a}}{}^{-1}_{sj} \frac{\partial \mathbf{S}_{\mathrm{e}}}{\partial \mathbf{C}_{\mathrm{e}}}_{rstu} \mathbf{F}_{\mathrm{a}}{}^{-1}_{tk} \mathbf{F}_{\mathrm{a}}{}^{-1}_{lu} \Bigg\}.
\end{aligned} \tag{S19}$$

In the above equation, the elastic stress derivative is computed to

$$\begin{aligned}
\frac{\partial \mathbf{S}_{\mathrm{e}}}{\partial \mathbf{C}_{\mathrm{e}}} = \tfrac{1}{2}\gamma \Big[& \delta_0 \tilde{\mathbf{L}} \otimes \tilde{\mathbf{L}} + \delta_2 \left(\mathbf{C}_{\mathrm{e}}^{-1} \tilde{\mathbf{L}} \mathbf{C}_{\mathrm{e}}^{-1} \otimes \mathbf{C}_{\mathrm{e}}^{-1} \tilde{\mathbf{L}} \mathbf{C}_{\mathrm{e}}^{-1} \right) + \delta_3 \left(\mathbf{C}_{\mathrm{e}}^{-1} \otimes \mathbf{C}_{\mathrm{e}}^{-1} \tilde{\mathbf{L}} \mathbf{C}_{\mathrm{e}}^{-1} + \mathbf{C}_{\mathrm{e}}^{-1} \tilde{\mathbf{L}} \mathbf{C}_{\mathrm{e}}^{-1} \otimes \mathbf{C}_{\mathrm{e}}^{-1} \right) \\
& + \delta_4 \mathbf{C}_{\mathrm{e}}^{-1} \otimes \mathbf{C}_{\mathrm{e}}^{-1} + \delta_5 \frac{\partial \mathbf{C}_{\mathrm{e}}^{-1}}{\partial \mathbf{C}_{\mathrm{e}}} + \delta_6 \frac{\partial \mathbf{C}_{\mathrm{e}}^{-1} \tilde{\mathbf{L}} \mathbf{C}_{\mathrm{e}}^{-1}}{\partial \mathbf{C}_{\mathrm{e}}} \Big]
\end{aligned} \tag{S20}$$

with the scalar pre-factors

$$\begin{aligned}
\delta_0 &= \alpha \mathrm{e}^{\alpha(\tilde{I}_{\mathrm{e}}-1)}, \qquad \delta_2 = \beta \mathrm{e}^{\beta(\tilde{J}_{\mathrm{e}}-1)} \det(\mathbf{C}_{\mathrm{e}})^2, \qquad \delta_3 = -(\beta \tilde{J}_{\mathrm{e}} + 1) \mathrm{e}^{\beta(\tilde{J}_{\mathrm{e}}-1)} \det(\mathbf{C}_{\mathrm{e}}), \\
\delta_4 &= (\beta \tilde{J}_{\mathrm{e}} + 1) \mathrm{e}^{\beta(\tilde{J}_{\mathrm{e}}-1)}, \qquad \delta_5 = \tilde{J}_{\mathrm{e}} \mathrm{e}^{\beta(\tilde{J}_{\mathrm{e}}-1)}, \qquad \delta_6 = -\mathrm{e}^{\beta(\tilde{J}_{\mathrm{e}}-1)} \det(\mathbf{C}_{\mathrm{e}}).
\end{aligned}$$

The second derivative of the activation level ω_{a} in Eq. (S19) reads

$$\frac{\partial^2 \omega_{\mathrm{a}}}{\partial \mathbf{C}^2} = \frac{1}{4\lambda^3} \left(\lambda \frac{\partial^2 \omega_{\mathrm{a}}}{\partial \lambda^2} - \frac{\partial \omega_{\mathrm{a}}}{\partial \lambda} \right) \mathbf{M} \otimes \mathbf{M}. \tag{S21}$$

Due to the implicit formulation of the activation level, the derivatives $\frac{\partial \omega_a}{\partial \lambda}$ and $\frac{\partial^2 \omega_a}{\partial \lambda^2}$ are approximated by a central differences scheme. Further, the derivatives of the active deformation gradient and its inverse are given by

$$\frac{\partial^2 \mathbf{F}_a}{\partial \omega_a^2} = -\tfrac{3}{4}(1-\omega_a)^{-\frac{5}{2}}(\mathbf{I}-\mathbf{M}), \tag{S22}$$

$$\frac{\partial \mathbf{F}_a^{-1}}{\partial \omega_a} = -\mathbf{F}_a{}^{-1}\frac{\partial \mathbf{F}_a}{\partial \omega_a}\mathbf{F}_a{}^{-1}, \tag{S23}$$

$$\frac{\partial^2 \mathbf{F}_a^{-1}}{\partial \omega_a^2} = -\left(2\mathbf{F}_a{}^{-1}\frac{\partial \mathbf{F}_a}{\partial \omega_a}\frac{\partial \mathbf{F}_a^{-1}}{\partial \omega_a} + \mathbf{F}_a{}^{-1}\frac{\partial^2 \mathbf{F}_a}{\partial \omega_a^2}\mathbf{F}_a{}^{-1}\right). \tag{S24}$$

S1.4 Modified generalized active strain approach (GASAM)

The material tangent for the GASAM-model is computed by adding the additional term $\mathbb{C}_{\omega_a}$ to the elasticity tensor $\mathbb{C}$ in Eq. (S9). Based on the additional stress contribution $\mathbf{S}_{\omega_a}$ in Eq. (25), $\mathbb{C}_{\omega_a}$ reads

$$\mathbb{C}_{\omega_a} = 2\frac{\partial \mathbf{S}_{\omega_a}}{\partial \mathbf{C}} = \tfrac{\gamma}{4}e^{\alpha(\tilde{I}-1)}\left(\left(\alpha\tilde{I}' + \frac{1}{\lambda}\right)\frac{\partial \omega_a}{\partial \lambda} + \frac{\partial^2 \omega_a}{\partial \lambda^2}\right)\mathbf{M}\otimes\mathbf{M} \tag{S25}$$

where

$$\frac{\partial^2 \omega_a}{\partial \lambda^2} = \frac{1}{\alpha\lambda^2}\left(\frac{6}{\lambda^2}\ln\phi + \frac{1}{\phi}\left(\phi'' - \frac{1}{\phi}\phi'^2 - \frac{4}{\lambda}\phi'\right)\right) \tag{S26}$$

and

$$\phi'' = \frac{\partial^2 \phi}{\partial \lambda^2} = \tfrac{4\alpha^2}{\gamma}e^{\alpha(1-\tilde{I}_p)}P_{opt}f_t^{tanh}\left(\left(\alpha\tilde{I}_p'^2 - \tilde{I}_p''\right)\int_{\lambda_{min}}^{\lambda} f_\xi(\tilde{\lambda})d\tilde{\lambda} - 2f_\xi\tilde{I}_p' + \tfrac{1}{\alpha}\frac{df_\xi}{d\lambda}\right). \tag{S27}$$

S2 Parameter identification: Details on the optimization algorithm setup

To fit the material parameters to the experimental data, we solve a least-squares minimization problem using the Trust Region Reflective algorithm [3]. The bounds and initial guesses for the optimization parameters applied in this study are listed in Table S1.

Table S1 Bounds and initial guesses for the optimization parameters. For brevity, the bounds and initial guesses applied to the ASA-, GASA-, and GASAM-models are listed together. Note that while the ASA- and GASA-models use the parameter N_a, the GASAM-model uses the parameter P_{opt}.

ASE-model

Parameter	Unit	Bounds		Initial guess
		Lower	Upper	
G_1	kPa	0.1	∞	5
G_2	kPa	0.05	∞	5
D_1	-	0.05	∞	1
D_2	-	0.05	∞	1
λ_{opt}	-	1.0	1.3	1.15
λ_*	-	1.4	2.0	1.7
σ_{max}	kPa	0.1	∞	10
μ	kPa	0	10	0.1

ASA-, GASA-, and GASAM-models

Parameter	Unit	Bounds		Initial guess
		Lower	Upper	
α	-	0	∞	1
β	-	0	∞	1
γ	kPa	0	∞	1
ω_0	-	0.0	1.0	0.5
P_{opt}	kPa	0	100	70
N_a	mm^{-2}	0	0.7	0.5
λ_{min}	-	0.35	1.0	0.6
λ_{opt}	-	1.0	1.3	1.15

S3 Fusifom muscle contraction simulations: Visualizations of von Mises stress

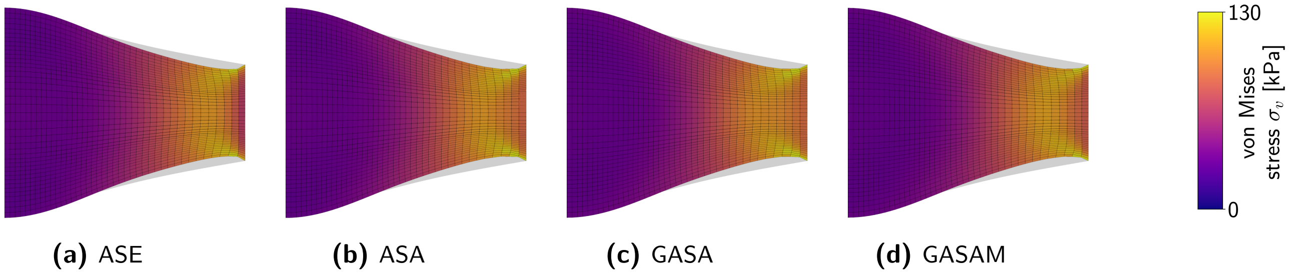

Figure S1 Von Mises stress σ_v in the axial cross section of the fusiform muscle ($n = 4$) for an isometric contraction in the tetanized state at $t = 0.15$ s. The initial configuration is displayed in grey. For symmetry reasons, only half the muscle is visualized.

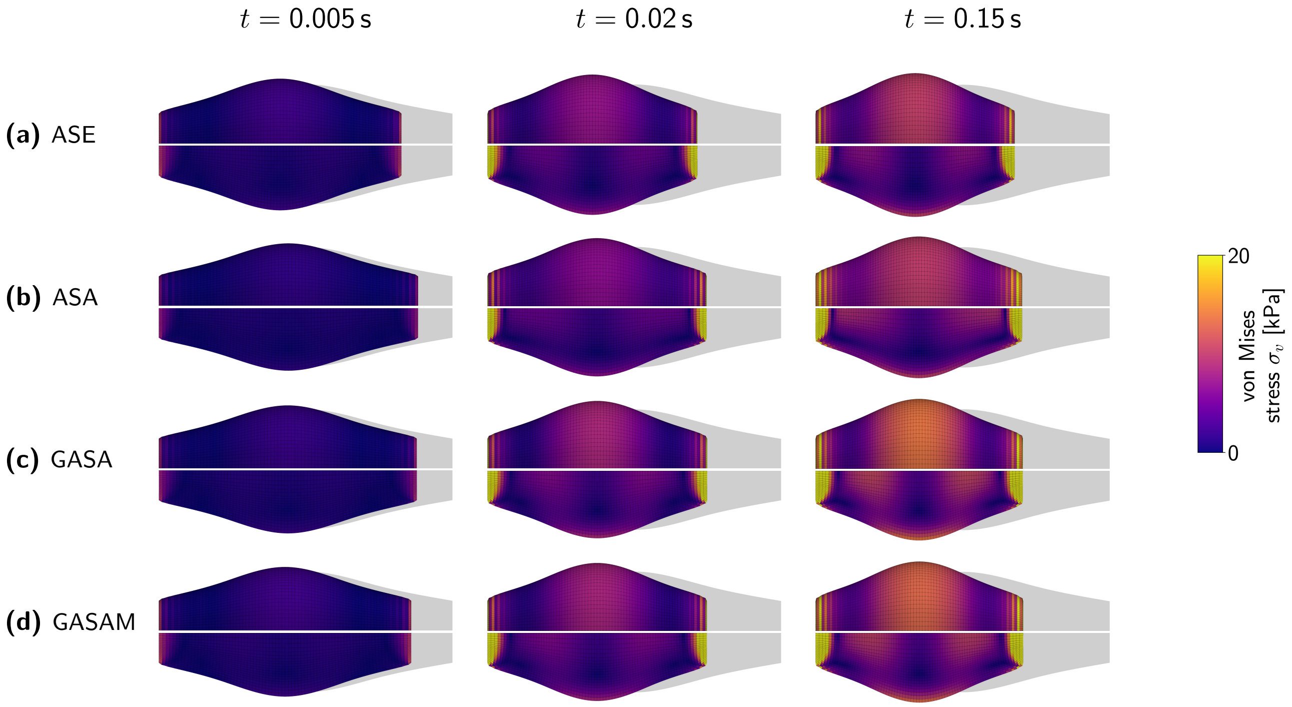

Figure S2 Von Mises stress σ_v for a free contraction of the fusiform muscle ($n = 4$) at selected times. Results are visualized on the surface (top) and in the axial cross-section (bottom) in comparison to the initial configuration (grey).

References

[1] G. A. Holzapfel, *Nonlinear solid mechanics: A continuum approach for engineering*, 1st ed. Chichester: Wiley, 2000.

[2] J. Weickenmeier, M. Itskov, E Mazza, and M. Jabareen, "A physically motivated constitutive model for 3D numerical simulation of skeletal muscles," *International Journal for Numerical Methods in Biomedical Engineering*, vol. 30, no. 5, pp. 545–562, 2014, DOI: 10.1002/cnm.2618.

[3] M. A. Branch, T. F. Coleman, and Y. Li, "A subspace, interior, and conjugate gradient method for large-scale bound-constrained minimization problems," *SIAM Journal on Scientific Computing*, vol. 21, no. 1, pp. 1–23, 1999, DOI: 10.1137/S1064827595289108.
